# Optical imaging of ovarian cancer using a matrix metalloproteinase-3-sensitive near-infrared fluorescent probe

**DOI:** 10.1371/journal.pone.0192047

**Published:** 2018-02-01

**Authors:** Kuo-Hwa Wang, Yung-Ming Wang, Li-Hsuan Chiu, Tze-Chien Chen, Yu-Hui Tsai, Chun S. Zuo, Kuan-Chou Chen, Chun Austin Changou, Wen-Fu T. Lai

**Affiliations:** 1 Graduate Institute of Clinical Medicine, College of Medicine, Taipei Medical University, Taipei, ROC; 2 Department of Obstetrics and Gynecology, Chung Kang branch, Cheng Ching Hospital, Taichung, Taiwan, ROC; 3 Department of Biological Science and Technology, National Chiao Tung University, Hsinchu, Taiwan, ROC; 4 McLean Imaging Center, McLean Hospital, Harvard Medical School, Belmont, MA, United States of America; 5 Department of Obstetrics and Gynecology, Mackay Memorial Hospital, Taipei, ROC; 6 Department of Research, Taipei Medical University/Shuang-Ho Hospital, New Taipei City, Taiwan, ROC; 7 Department of Urology, Taipei Medical University/Shuang-Ho Hospital, New Taipei City, Taiwan, ROC; 8 The Ph.D. Program for Cancer Biology and Drug Discovery, Center for Translational Medicine, Taipei Medical University, Taipei, ROC; 9 Department of Dentistry, Taipei Medical University/Shuang-Ho Hospital, New Taipei City, Taiwan, ROC; Stanford University, UNITED STATES

## Abstract

Epithelial ovarian cancer (EOC) is the seventh most common cancer among women worldwide. The 5-year survival rate for women with EOC is only 30%-50%, which is largely due to the typically late diagnosis of this condition. EOC is difficult to detect in its early stage because of its asymptomatic nature. Recently, near-infrared fluorescent (NIRF) imaging has been developed as a potential tool for detecting EOC at the molecular level. In this study, a NIRF-sensitive probe was designed to detect matrix metalloproteinase (MMP) activity in ovarian cancer cells. A cyanine fluorochrome was conjugated to the amino terminus of a peptide substrate with enzymatic specificity for MMP-3. To analyze the novel MMP-3 probe, an *in vivo* EOC model was established by subcutaneously implanting SKOV3 cells, a serous-type EOC cell line, in mice. This novel MMP-3-sensitive probe specifically reacted with only the active MMP-3 enzyme, resulting in a significantly enhanced NIRF emission intensity. Histological analysis demonstrated that MMP-3 expression and activity were enhanced in the stromal cells surrounding the ovarian cancer cells. These studies establish a molecular imaging reporter for diagnosing early-stage EOC. Additional studies are required to confirm the early-stage activity of MMP-3 in EOC and its diagnostic and prognostic significance.

## Introduction

Epithelial ovarian cancer (EOC) is the fifth leading cause of cancer-related death among women [1, 2]. Approximately 239,000 cases were recorded in 2012, accounting for nearly 4% of all new cancer cases among women worldwide (2% overall). The overall 5-year survival rate for EOC is below 45% [1], largely because of the considerable difficulty involved in detecting the disease at early stages. Consequently, approximately 75% of ovarian cancers are diagnosed at stages III or IV.

Cancer metastasis requires the dissociation of cancer cells from the primary lesion site, local tissue invasion, angiogenesis, blood or lymphatic system invasion, and seeding at distant lesion sites [3, 4]. Matrix metalloproteinases (MMPs) play a critical role in facilitating cancer invasion and metastasis [5] through the proteolysis of extracellular matrix (ECM) and nonmatrix components [6]. Specific MMPs such as MMP-3, -7, and -9 promote cancer metastasis [7–9] and are modulated by WNT signaling [10]. Among them, MMP-3 (stromelysin-1) is overexpressed in various tumor types [11], and its activation has been reported in ovarian cancer [12, 13]. Although several MMPs have been studied in ovarian cancer [7, 14, 15], few studies have focused on MMP-3 in EOC. During tumorigenesis, MMP-3 is a key candidate for regulating tumor-host interactions [16], which facilitate early-stage breast carcinogenesis [17]. However, MMP-3 also inhibits tumor progression in breast cancer and skin squamous cell carcinoma, studies have reported increased tumor growth rates and higher proliferative indices in MMP-3-null mice [16, 18]. In addition, MMP-3 activity may reflect a wound-healing response of the associated tumor stroma as MMP-3 attempts to reconcile the tumor cells back into a normal tissue structure and to limit cancer cell invasion [16]. Thus, the analysis of early-stage MMP expression, including MMP-3 expression, may serve as a potential molecular target for imaging prior to cancer metastasis and progression, especially given the importance of the tumor stroma in ovarian cancer progression [19].

Molecular imaging is a novel tool for the *in vivo* characterization of biologic processes at the cellular and molecular level [20, 21]. Contrary to traditional diagnostic imaging techniques such as computed tomography (CT), magnetic resonance imaging (MRI), and ultrasound, molecular imaging enables the analysis of molecular mechanisms underlying disease abnormalities. Various molecular imaging platforms have been employed, including optical imaging (both fluorescence and bioluminescence), single-photon emission computed tomography (SPECT), and positron emission tomography (PET), with substrate-based and activity-based probes [22–24].

Near-infrared fluorescence (NIRF) imaging is an attractive modality for early cancer detection with high sensitivity and multidetection capability. NIRF probes are ideal candidates for the targeted imaging of cancer tissues, especially given their convenient modification through conjugation with the moieties of interest [25]. Moreover, NIRF imaging can be combined with other imaging modalities, and nanoparticles loaded with NIRF dyes and anticancer agents have the potential to combine; exploit imaging and therapeutic functions to achieve the ultimate goal of simultaneous diagnosis and treatment [25]. NIRF has been employed to detect cathepsin [26], lysophosphatidic acid [27], and MMP enzymatic activity [28]. Other probes have been designed to directly bind to HER2 [29] or *α*_v_*β*_3_ receptor [30] to target specific cancer cell types. Activity-based signals can be detected by NIRF-quenched probes, which can shift their optical properties after protease cleavage. Upon cleavage, the fluorochrome and the quencher separate, resulting in an enhanced fluorescence signal.

Given that MMP-3 is associated with tumor-activated stromal cells [31, 32], a novel MMP-3-sensitive NIRF probe has been designed in our laboratory to detect stromal cell activation by early-stage EOC. A NIR fluorochrome was conjugated to the amino terminus of an MMP-3-specific peptide substrate. To analyze the novel MMP-3 probe, an *in vivo* EOC model was established by subcutaneously implanting SKOV3 cells, a serous-type EOC cell line, in mice. This present study may provide data for delineating the role of MMP-3 expression in EOC disease progression and its clinical utility in early-stage EOC detection.

## Materials and methods

### Reagents

Methoxy-polyethylene glycol was obtained from Fluka Chemical Co. (Buchs, St. Gallen, Switzerland) and *N*-hydroxysuccinimide (NHS) from Aldrich Chemical Co. (St. Louis, MO, USA). All other reagents and solvents were also purchased from Aldrich. Absorbance and fluorescence spectra were measured on a U-3010 spectrophotometer (Hitachi, Tokyo, Japan) and an F-4500 fluorophotometer (Hitachi, Tokyo, Japan), respectively. Proton nuclear magnetic resonance (NMR) spectra were obtained in CDCl_3_ in a 400 MHz Fourier-transform-NMR spectrometer and the functional groups were recorded using a Perkin Elmer FT-IR system 2000 (Waltham, MA, USA).

### Synthesis of the MMP-3-sensitive probe

The peptide-protected graft copolymer (PGC) was synthesized from a precursor, methoxy-polyethylene glycol (MPEG) nitrile.

#### Synthesis of MPEG nitrile

A mixture MPEG of molecular weight 5000 Da (25 g, 5mM), distilled water (25 mL) and potassium hydroxide (0.5 g) was cooled to 0°C-5°C in an ice bath. Acrylonitrile (4.3 mL) was added slowly, and the solution was stirred for 2.5 h at 0°C -5°C. The pH of the solution was adjusted to 7 by adding sodium phosphate. The product was extracted with dichloromethane (200, 70, and 50 mL). The organic layer was dried over magnesium sulfate. The solution was concentrated, and the product was precipitated through adding diethylether. The yield of MPEG nitrile was 23.5 g (93%) with the following characteristics: IR, 2360 cm^-1^ (-CN); NMR (CDCl_3_), 2.61 ppm (t, 2H, -C*H*_*2*_CN), 3.3 ppm (s, -OC*H*_*3*_), and 3.5–3.8 ppm (m, -OC*H*_*2*_C*H*_*2*_O-).

#### Synthesis of MPEG amide

A mixture MPEG nitrile (23.5 g, 4.7mM) and concentrated hydrochloric acid (98 mL) was stirred at room temperature for 48 h. The solution was diluted with 1 L of water and extracted with dichloromethane (200, 150, and 100 mL). The combined organic layer extracts were washed twice with water, dried over sodium sulfate, filtered, and concentrated to dryness through rotation evaporation. The yield of MPEG amide was 21.5 g (91%) with the following characteristics: IR, (3424cm^-1^, -NH_2_) and (1638 cm^-1^, -CONH_2_); NMR CDCl_3_, 2.47 ppm (t, 2H, -C*H*_*2*_-CONH_2_), 3.3 ppm (s, -OC*H*_*3*_), and 3.5–3.8 ppm (m, -OC*H*_*2*_C*H*_*2*_O-).

#### Synthesis of MPEG propionic acid

MPEG amide (16 g, 3.2mM) was dissolved in 1000 mL of distilled water; 100 g of potassium hydroxide was added, and the solution was stirred for 22 h at room temperature. Sodium chloride (150 g) was added, and the solution was extracted with dichloromethane (150 mL × 3). The combined organic extracts were washed with 5% oxalic acid and water (twice), and dried over sodium sulfate. The solution was concentrated, and the product was precipitated through adding diethylether. The product, MPEG propionic acid, was collected by filtration and dried over vacuum. The yield of MPEG propionic acid was 14.0 g (87.5%) with the following characteristics: IR, (3453 cm^-1^, -OH) and NMR (CDCl_3_), 2.57 ppm (t, 2H, -C*H*_*2*_-COOH), 3.3 ppm (s, -OC*H*_*3*_), and 3.5–3.8 ppm (m, -OC*H*_*2*_C*H*_*2*_O-).

#### Synthesis of MPEG succinimidyl propionic acid (MPEG-SPA)

MPEG propionic acid (3.4 g, 1mM) was dissolved in dichlorormethane (20 mL), and *N*-hydroxysuccinimide (0.24 g, 2.1mM) was added. The solution was cooled to 0°C, and a solution of dicyclohexylcarbodiimide (0.43g, 2.1mM) in 4 mL dichloromethane was added dropwise and stirred at room temperature overnight. The reaction mixture was filtered, concentrated, and precipitated by addition to diethylether. The yield of MPEG-SPA was 3.3 g (95%) with the following characteristics: NMR (CDCl_3_), 2.81 ppm (s, 4H, NHS), 2.92 ppm (t, 2H, -C*H*_*2*_-COO-), 3.3 ppm (s, -OC*H*_*3*_), and 3.5–3.8 ppm (m, -OC*H*_*2*_C*H*_*2*_O-). (S1 Scheme)

#### Synthesis of PGC

PGC was synthesized using MPEG-SPA (397.5 mg, 79.5μM), which was added slowly to an ice-cold solution of poly-L-lysine hydrobromide (14.6 kDa; 50 mg, 3.4μM) in sodium bicarbonate (0.1M, pH 8.0, 12.5 mL). The pH was adjusted to 7.7 by using sodium hydroxide, and the mixture was stirred at room temperature for 3 h. The solution was then extensively ultrafitered (cutoff molecular weight of 3kDa) to remove small-molecular-weight byproducts. PGC was recovered through lyophilization (73.6 kDa; 50.8 mg, 99%), and the available amino groups for chemical conjugation were quantified using a 2,4,6-trinitrobenzene sulfonic acid (TNBS) assay. There were 51.7 ± 0.2 free amino groups per PGC molecule.

#### Synthesis of iodoacetylate PGC

PGC (1 mg, 0.1μM) in 200 μL of 50mM NaHCO_3_ was iodoacetylated through reaction with an excess of iodoacetic anhydride (3.5 mg, 10μmol) in 100 μL of DMF for 3 h. Ultrafitration (cutoff of 3 kDa) was used to separate the product from the excess reagent, byproducts, and solvent. No free amino group was detected using the TNBS assay.

#### Synthesis of the MMP-3 peptide

A peptide substrate of MMP-3 (EC 3.4.24.17) was synthesized on an automatic synthesizer (PS3, Rainin Instrument Co.) through an Fmoc chemistry by using benzotriazole-1-yl-oxy-tris-pyrrolidino-phosphonium hexafluoro-phosphate (PyBOP) as the activating agent. The sequences were modified from previous studies [33, 34]. The sequences are Gly-Pro-Lys-Pro-Tyr-*Arg-Ser*-Trp-Met-Lys(FITC)-Cys-NH_2_ (GPKPY*RS*WMK(FITC)C-NH_2_) for the stromelysine-1-sensitive peptide substrate and Gly-Pro-Lys-Pro-Tyr-Arg-Pro-Trp-Met-Lys(FITC)-Cys-NH_2_ (GPKPYRPWMK(FITC)C-NH_2_) for the scrambled control peptide (S1 Fig). ESI-TOF-MS (M+H)^+^ analysis revealed the following characteristics: stromelysine-1 peptide substrate, 1709.8 (calculated) and 1710 (found); control peptide, 1719.7 (calculated) and 1720 (found).

#### Specificity of the peptide substrate

The stromelysine-1 peptide substrate or control peptide (1 mg, 0.58 μmol) was incubated with 1 unit of MMP-3 for overnight reaction. MMP-3 was supplied in a 50mM Tris buffer solution (pH 7.4) containing 0.5M sodium chloride, 10mM calcium chloride, 0.05% Brij-35 and 0.02% sodium azide.

#### Synthesis of peptide-PGC

The iodoacetylated PGC was coupled to 3.2 mg of stromelysine-1 peptide substrate or 3.2 mg of control peptide through a thiol-specific reaction in 200 μL of acetonitrile and 200 μL of 0.1M sodium acetate (pH 6.5) for 3 h. Excess peptide and byproducts were separated from the peptide-PGC conjugate through sephadex G-25 size-exclusion chromatography. The peptide-PGC conjugate was determined by measuring the fluorescence absorption at 494nm divided by initial available amino groups. A total of 13.1 ± 0.2 stromelysine-1 peptide substrate chains or 13.8 ± 0.2 control peptide chains were coupled per PGC molecule. To conjugate Cy5.5 dye with the peptide-PGC, 1μM monoactivated near-infrared Cy5.5 (Amersham-Pharmacia, Piscataway, NJ, USA) was reacted with the N-terminus of the peptide‑PGC (0.5 mg) in 2 mL of 50mM NaHCO_3_. The reaction mixture was incubated at room temperature for 1 h, and products were purified using ultrafiltration with a 3kDa cutoff (S2 Fig). HPLC chromatogram of stromelysine-1 peptide substrate and control peptide substrate were shown (S3 and S4 Figs), as well as the photochemical data of the final probes (S5, S6, S7 and S8 Figs).

### MMP-3 digestion assay

An MMP-3 digestion assay was applied to examine the *in vitro* reaction of the MMP-3-sensitive probe and MMP-3 enzyme through NIRF imaging. Briefly, MMP-3 (Sigma, M1677) was activated with P-aminophenylmercuric acetate (APMA) for 1 h at 37°C. After activation, an equal volume of the MMP-3-sensitive probe was mixed with the activated MMP-3/APMA solution. To prepare the solutions for the control groups, equal volumes of the MMP-3-sensitive probe was mixed with an MMP-3 solution without the activation or blank reaction buffer. All final solutions were incubated at 37°C for 30 min before being subjected to NIRF examination and quantification. For *in vitro* fluorescence quantification, six samples were measured in triplicate. Optical imaging of MMP-3 NIRF was performed using an optical imaging (IVIS-200 Series; Caliper, MA). Excitation and emission bandpass filters of 610-650nm and 670-700nm, respectively were employed. Both white light and NIRF images were detected using a Peltier 16-bit charge-coupled device (CCD) camera (PCO GmbH, Gottingen, Germany, −90°C cooled).

### Coculture of SKOV3 and WS1 cells

Ovarian cancer cell line, SKOV3, and fibroblast cell line, WS1, were purchased from ATCC (Manassas, VA, USA). The cells were grown and expanded in McCOY’s 5A medium containing 10% fetal bovine serum (FBS), 100 units/mL penicillin, and 100 μg/mL streptomycin and amphotericin B in an incubator at 37°C with 5% CO_2_. The cell model was divided into six groups: 2 × 10^4^ SKOV3 cells alone, 2 × 10^4^ SKOV3 cells cocultured with 2 × 10^4^ WS1 cells, 2 × 10^4^ SKOV3 cells cocultured with 6 × 10^4^ WS1 cells, 6 × 10^4^ SKOV3 cells alone, 6 × 10^4^ SKOV3 cocultured with 2 × 10^4^ WS1 cells, and 6 × 10^4^ SKOV3 cocultured with 6 × 10^4^ WS1 cells. Cells were cocultured in 24-well dishes in 1 mL of McCOY’s 5A medium with 10% FBS for 3 days.

### Cell imaging

To examine the specificity of the MMP-3-sensitive probe in detecting enzymatic activity in a cell culture model, SKOV3 cells, WS1 cells, and cocultured SKOV3 and WS1 cells (3 days) were treated with the MMP-3 NIRF probes for 30 min prior to imaging and detecting the enzymatic activity. The cells were washed three times with phosphate-buffered saline (PBS) and then subjected to confocal fluorescence microscopy (LEXT OLS4100, Olympus, Tokyo, Japan). The bandpass excitation filter was at 633nm, and the longpass emission filter was 670nm (Omega Optical, Brattleboro, VT, USA). The fluorescence images were digitally captured using a CCD-SPOT RT digital camera and compiled using Adobe Photoshop™ software (v7.0) (Softonic International S.A., Barcelona, Spain).

### Analysis of MMP-2, MMP-3 and MMP-9 expression in cocultured SKOV3 and WS1 cells

To analyze various MMP expression levels, SKOV3, WS1, and cocultured SKOV3 and WS1 cells (1:1) were seeded at 1.5 × 10^4^/well in 24-well plates. The supernatant was collected from each group at 1, 2, 4, 6, and 8 days to quantify the total expression levels of MMP-2, MMP-3 and MMP-9 through enzyme-linked immunosorbent assay (ELISA) using Abcam MMP human ELISA kits (Cambridge, MA, USA). The relative cell number was determined using a WST-1 cell proliferation reagent (Sigma-Aldrich, 96992).

### Imaging of MMP-3 NIRF in an in vivo EOC model

All nude mice were obtained and housed at Taipei Medical University (TMU), under barrier conditions in a biosafety level III animal laboratory room. All animals were treated according to the National Institutes of Health Guidelines for the Care and Use of Laboratory Animals. All animal study procedures were reviewed and approved by the TMU Laboratory Animal Care Committee. Six-week-old female nude mice were subcutaneously inoculated with 5 × 10^6^ SKOV3 cells suspended in 200 μL of PBS. Experiments with tumor-bearing mice were performed 3–11 days after inoculation. *In vivo* NIRF imaging was performed using an IVIS-200 system with excitation and emission filters at 610–650 nm and 670–700 nm, respectively. Control and SKOV3 tumor-bearing mice (n = 3 in each group) were intravenously injected with 200 μL of 2nM MMP-3-sensitive probe via the tail vein after which they were anesthetized with isoflurane and subjected to imaging analysis. Fluorescent images were taken 2 hours after the injection at day 3, 5, 7, 11. The tumor size were measured 4mm x 8mm. The fluorescence intensity was measured in the tumor mass (ROI size = 4mm x 8mm) after adjusting for background intensity (non-tumor site tissue on the back of the mice) with same size of measurement. The relative fluorescent intensities were compared between groups. After imaging, the mice were euthanized by CO_2_ inhalation according to the Guidelines for the Care and Use of Laboratory Animals. The tumors, the peritumor areas, and lesion sites, which were analyzed through NIRF imaging, were excised for pathological confirmation and immunohistochemical analysis. A stability test was performed using non-tumor inoculated mice to observe baseline fluorescent intensity and probe distribution. For biodistribution evaluation, Individual organs were harvested and further evaluated for fluorescent intensity.

### Analysis of MMP-3 expression in SKOV3 tumors

Five-micron (5 μm) paraffin-embedded sections were deparaffinized and rehydrated. Antigens were retrieved by immersion in DECLERE (Cell Marque, Hot Springs, AR, USA) in moist heat for 15 min. Potential nonspecific binding sites were blocked with 5% normal goat or rabbit serum in PBS. After blocking, the sections are incubated with antibodies specific for MMP-3 (1:25; Santa Cruz Biotechnology, Santa Cruz, CA, USA). After three 5-min washes in PBS, the sections were incubated with biotin-conjugated IgG (Vector Laboratories, Burlingame, CA, USA). A vector-ABC streptavidin-peroxidase kit with benzidine substrate was used for color development. Following counterstaining with diluted hematoxylin, the images were scanned using Aperio Scanscope Console software (Informer Technologies, Inc., Shingle Springs, CA, USA). Sections that were not incubated with the primary antibody served as a negative control. For NIRF imaging, paraffin-embedded sections (without staining) were examined and photographed using a NIRF microscope with 615–675- and 695–790-nm band-pass filters at the Cy5.5 channel.

### Statistical analysis

MMP-3 concentrations, relative expression, and signal intensity (SI) were presented as mean and ±standard deviations (SDs). Independent t tests were performed to compare the differences between two groups, one-way ANOVA was performed to compare the differences between three or more groups, and the Bonferroni post-hoc test was used for multiple comparisons. Statistical analyses were performed using IBM SPSS version 22 for Windows (IBM Corp., Armond, NY, USA), with two-tailed *P*<0.05 indicating statistical significance.

## Results

### Characterization and *in vitro* imaging of the MMP-3-sensitive NIRF probe

**Fig 1:**
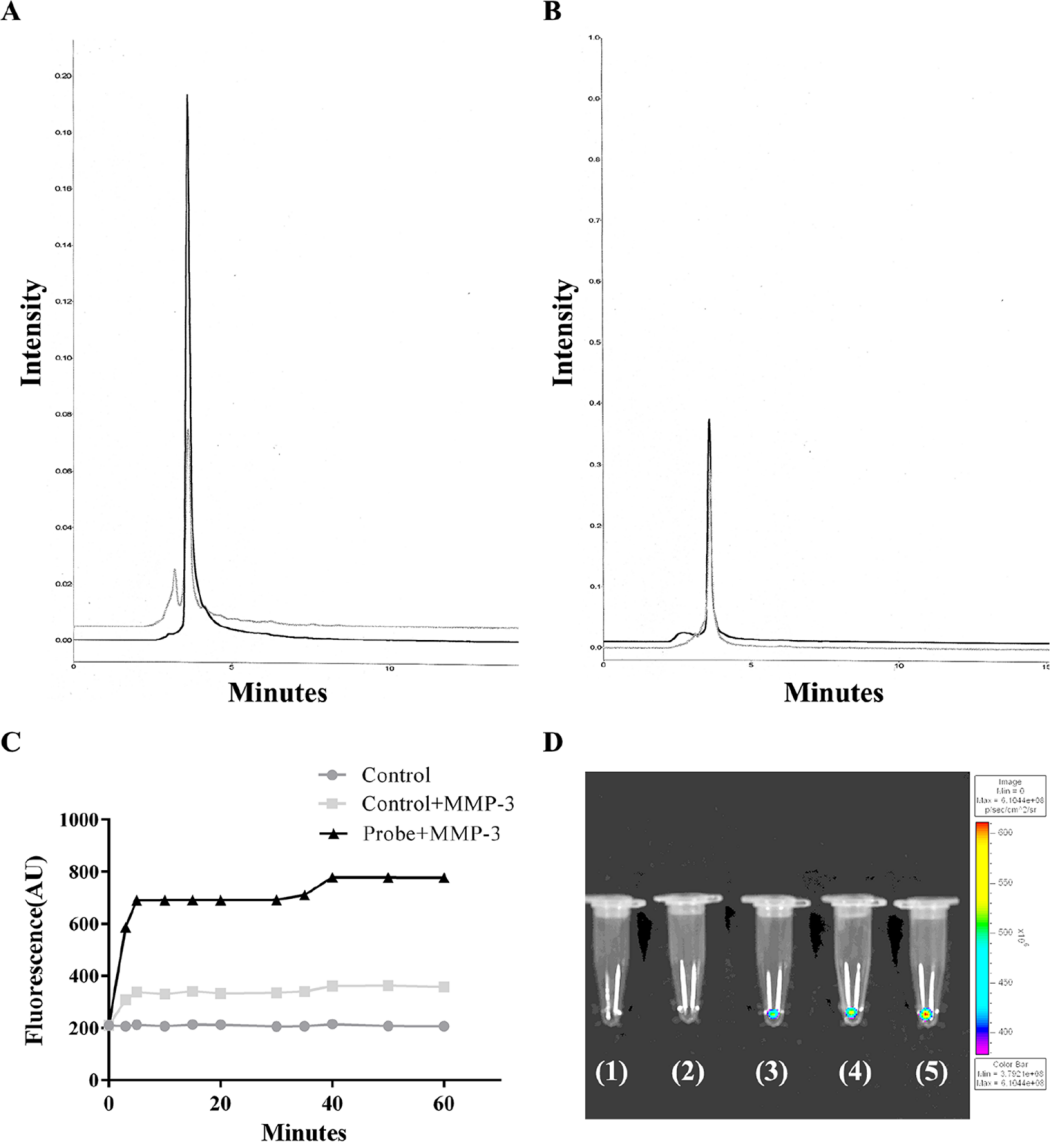
Characterization of the novel stromelysine-1 peptide substrate. HPLC chromatogram of the (A) stromelysine-1 peptide substrate (black) and their digested products by MMP-3(gray) as well as (B) the control peptide (black) and their digested products by MMP-3 (gray). (C) *In vitro* activation and inhibition of experimental and control NIRF probes; the probes (0.009 μM) were incubated with or without MMP-3 (1 unit) in 50 mM Tris buffer solution. (D) *In vitro* imaging of the MMP-3 NIRF probe activated by the MMP-3 enzyme. The manifest orange-yellow color was detected in the tubes containing the MMP-3 probe with activated MMP-3 (3–5) compared with the blank (1) and MMP-3 only (2) control tubes. A series of MMP-3 probes at 0.045 ng/μL (3), 0.09 ng/μL (4), and 0.18 ng/μL (5) with MMP-3 (0.1 ng/μL) resulted in increasing SIs of 3.85 ± 0.15, 4.51 ± 0.12, and 4.86 ± 0.17 respectively. Six samples in triplicate were measured. Optical imaging was performed at 610–650nm excitation and 670–700nm emission. HPLC, high-performance liquid chromatography; MMP, matrix metalloproteinase; NIRF, near-infrared fluorescence.

High-performance liquid chromatography (HPLC) elution chromatography confirmed one major cut of the stromelysine-1 peptide substrate in the presence of MMP-3 and the elution of two fragments (Fig 1A and 1B). For the specificity test of the peptide substrate, stromelysine-1 peptide substrate or control peptide (1 mg, 0.58 0·mol) was incubated with 1 unit of MMP-3 for overnight reaction. Because the scrambled control peptide was not a substrate of MMP-3, the peptide remained intact and eluted at the original time. Following the addition of MMP-3, the NIRF signal of the stromelysine-1 peptide substrate increased by 4-fold within 40 min, which was significantly greater than the 1-fold increase in NIRF signals observed for the control probe (Fig 1C).

To examine the specificity of the synthesized probe in detecting MMP-3 enzymatic activity, an *in vitro* enzyme digestion assay was employed. Although a manifest orange-yellow color was revealed in the tube of activated MMP-3, the other groups showed weak or no NIRF signals. The SI of the NIRF probes appeared in a dose-dependent manner. The data indicated that the MMP-3-sensitive probe can specifically react with only the active MMP-3 enzyme, resulting in a significantly enhanced intensity of NIRF emissions (Fig 1D).

### Sensitivity and specificity of MMP-3 probes in SKOV3 and WS1 cocultures

**Fig 2:**
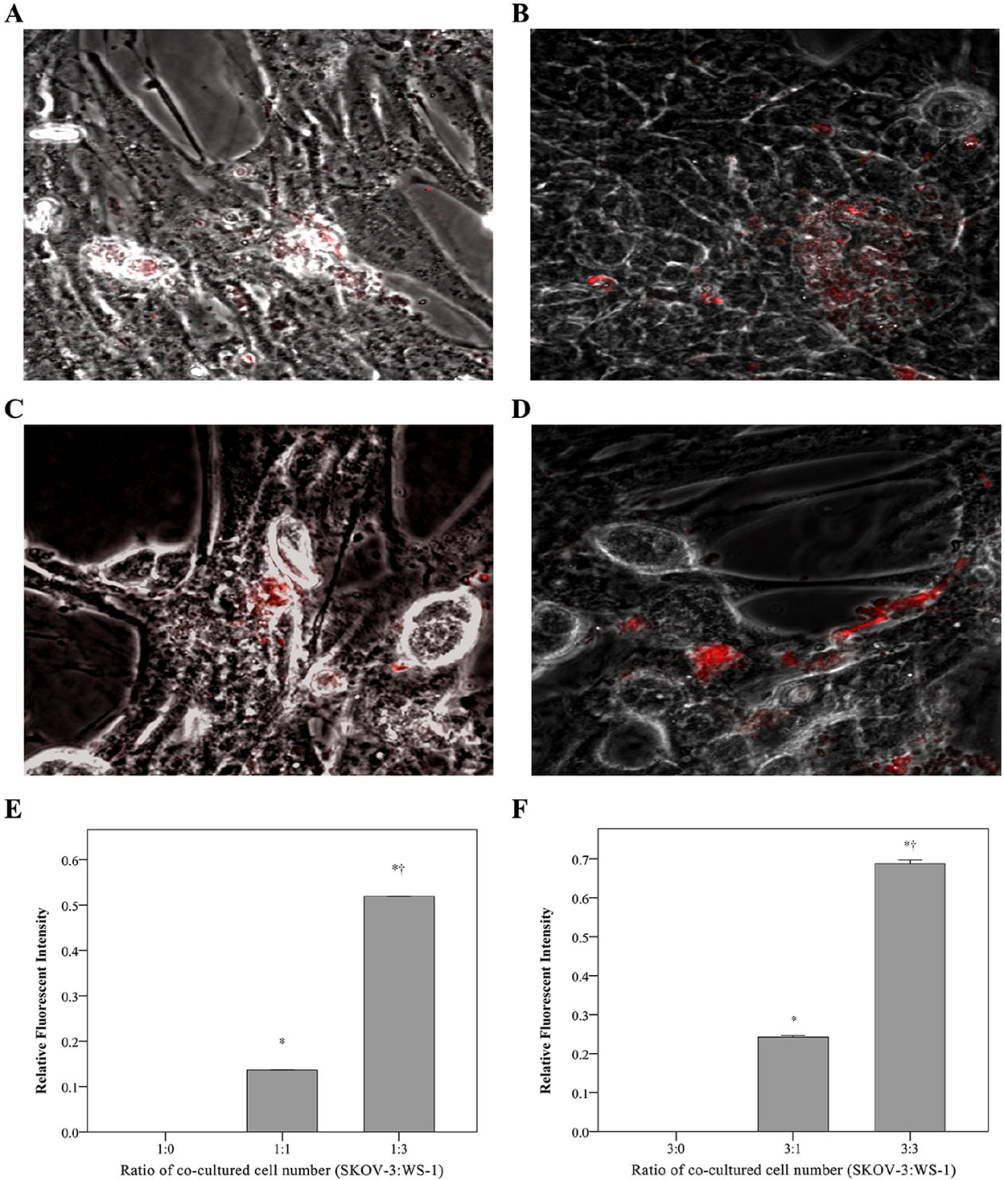
Detection of MMP-3 probes in SKOV3 and WS1 cell cocultures. (A–D) Detection of MMP-3 activity through NIRF imaging. (E, F) Quantification of the signal intensity. Either 1:1 or 3:1 (SKOV3:WS1) showed mild signal intensity (A,B), whereas 3:1 or 3:3 (SKOV3:WS1) exhibited remarkable signal intensity (C,D). The NIRF SI appeared undetectable in the culture of 2 × 10^4^, or 6 × 10^4^ SKOV3 cells alone. The mean MMP-3 concentration was significantly higher in cocultures with increasing WS1 cells than in SKOV3 cells cultured alone (*p*<0.001). The signal was significantly higher in the 1:3 (SKOV3:WS1) group than in the 1:1 group (0.52 vs. 0.14, respectively, *p*<0.001) (E). The mean MMP-3 concentration was significantly higher in the 3:1 and 3:3 (SKOV3:WS1) groups than in 3:0 group (0.24, 0.69 vs. 0, respectively; both *p*<0.001), and was significantly higher in the the 3:3 (SKOV3:WS1) cocultures than in the 3:1 cocultures (0.69 vs. 0.24, respectively; *p*<0.001) (F). MMP, matrix metalloproteinase; NIRF, near-infrared fluorescence; SI, signal intensity.

As shown in Fig 2A–2D, the SIs of the NIRF probes appeared to correlate with the MMP-3 levels in the cell culture media. The 2 × 10^4^ SKOV3 cells cocultured with 2 × 10^4^ WS1 cells, and 2 × 10^4^ SKOV3 cells cocultured with 6 × 10^4^ WS1 cells showed mild and remarkable signal intensity respectively (Fig 2A and 2C). The mean MMP-3 concentration was significantly higher in both of the coculture groups, whereas the 2 × 10^4^ SKOV3 cell groups had minimal florescence signals (*p*<0.001). The signal was significantly higher in the 1:3 (SKOV3:WS1) group than in the 1:1 group (0.52 vs. 0.14, respectively, *p*<0.001; Fig 2E).

The cell groups of 6 × 10^4^ SKOV3 cocultured with 2 × 10^4^ WS1, and 6 × 10^4^ SKOV3 cocultured with 6 × 10^4^ WS1 exhibited mild and remarkable signal intensity (Fig 2B and 2D). The mean MMP-3 concentration was significantly higher in the 3:1 and 3:3 (SKOV3:WS1) groups than in 3:0 group (0.24, 0.69 vs. 0, respectively; both *p*<0.001), and was significantly higher in the the 3:3 (SKOV3:WS1) cocultures than in the 3:1 cocultures (0.69 vs. 0.24, respectively; *p*<0.001; Fig 2F).

### MMP-2, MMP-3, and MMP-9 expression in SKOV3 and WS1 cell cocultures

**Fig 3:**
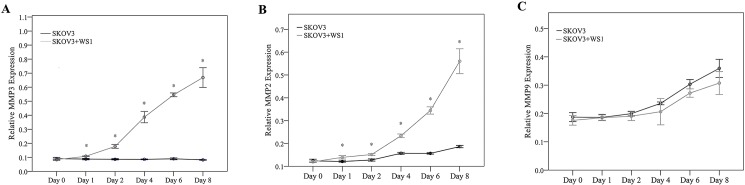
*In vitro* MMP expression in SKOV3 and WS1 cell cocultures. (A) MMP-3, (B) MMP-2, and (C) MMP-9 levels in the SKOV3 and WS1 cell cocultures (gray line) and SKOV3 cells cultured alone (black lines) were analyzed by ELISA. *p<0.05, significantly different compared with SKOV3 cell cultures. ELISA, enzyme-linked immunosorbent assay; MMP, matrix metalloproteinase.

The MMP-2, MMP-3, and MMP-9 expression levels were next examined in the cocultivation of SKOV3 and WS1 to mimic the interaction of ovarian cancer cell with stromal cells. The expression levels were determined in the cocultures and compared with the control group (SKOV3 alone). The mean relative MMP-3 expression level was significantly higher in the cocultures than in the SKOV3 control group at days 1 to 8 (all, *p*<0.001; Fig 3A).

The mean relative MMP-2 expression level was also significantly higher in the cocultures at days 1 to 8 (*p*≤0.004; Fig 3B). No significant differences were observed in the relative MMP-9 expression among the groups (all *p*>0.05; Fig 3C).

### MMP-3-sensitive probe detects SKOV3 cancer cells *in vivo*

**Fig 4:**
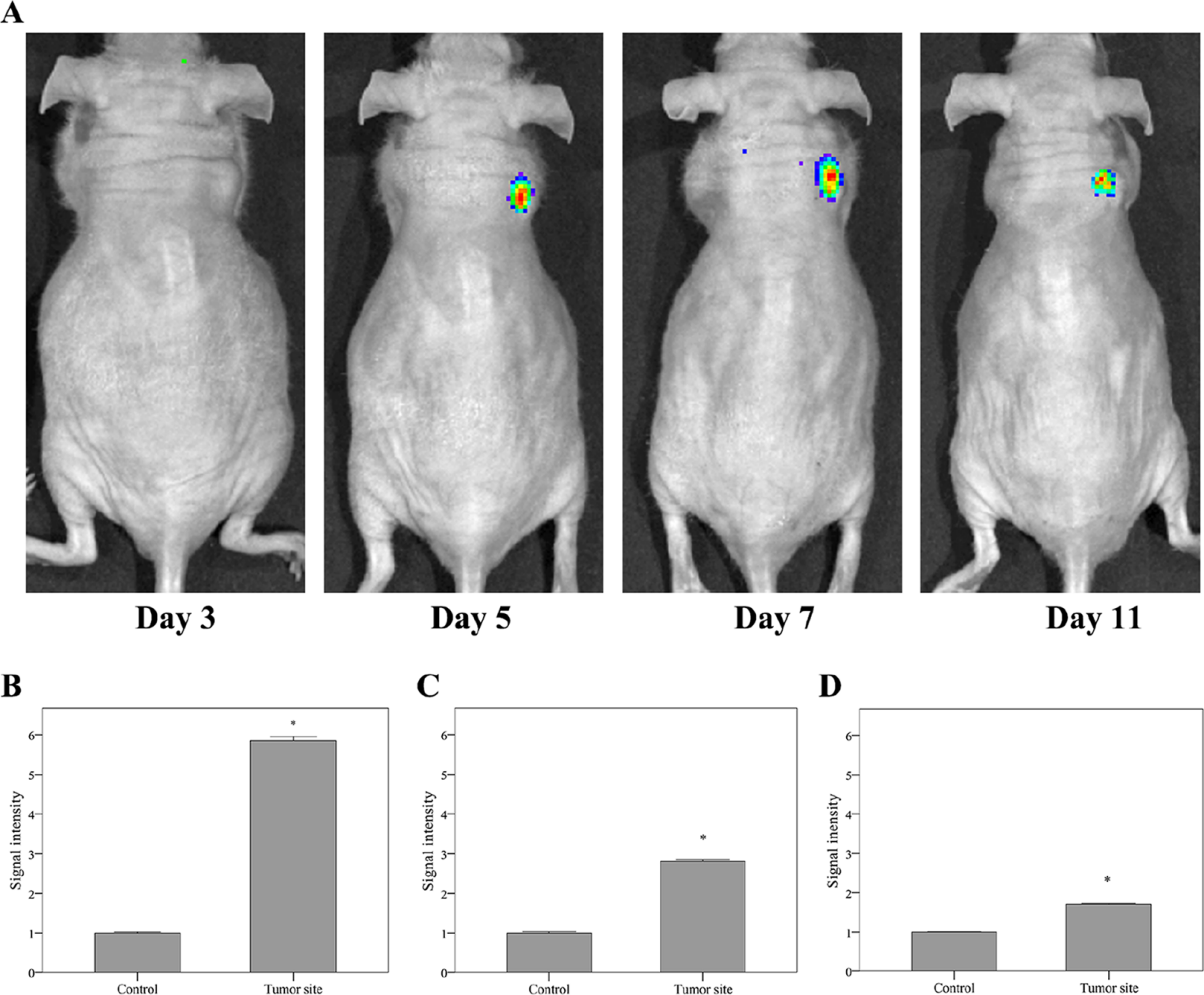
NIRF imaging of SKOV3 tumor masses *in vivo*. (A) NIRF imaging of the SKOV3 tumors at days 3, 5, 7, and 11. (B-D) The mean SI was significantly higher in the tumor than in the adjacent control tissue at days 5 (B), 7 (C), and 11 (D), *p<0.001, significantly different compared with the control tissue. NIRF, near-infrared fluorescence; SI, signal intensity.

Nude mice were inoculated with SKOV3 xenografts, which were imaged with the MMP-3-sensitive probe at days 3, 5, 7, and 11 postimplantation. As shown in Fig 4A, the NIRF signal was not detected in the tumor until day 5 postimplantation, at which time a 5-fold fluorescence intensity was observed compared with that detected at day 3 (Fig 4B). The NIRF signal was slightly decreased at days 7 and 11 (Fig 4C and 4D, respectively), suggesting that the MMP-3 activity peaked at day 5 and gradually decreased thereafter.

### MMP-3 activity and expression patterns in the SKOV3 tumor mass

**Fig 5:**
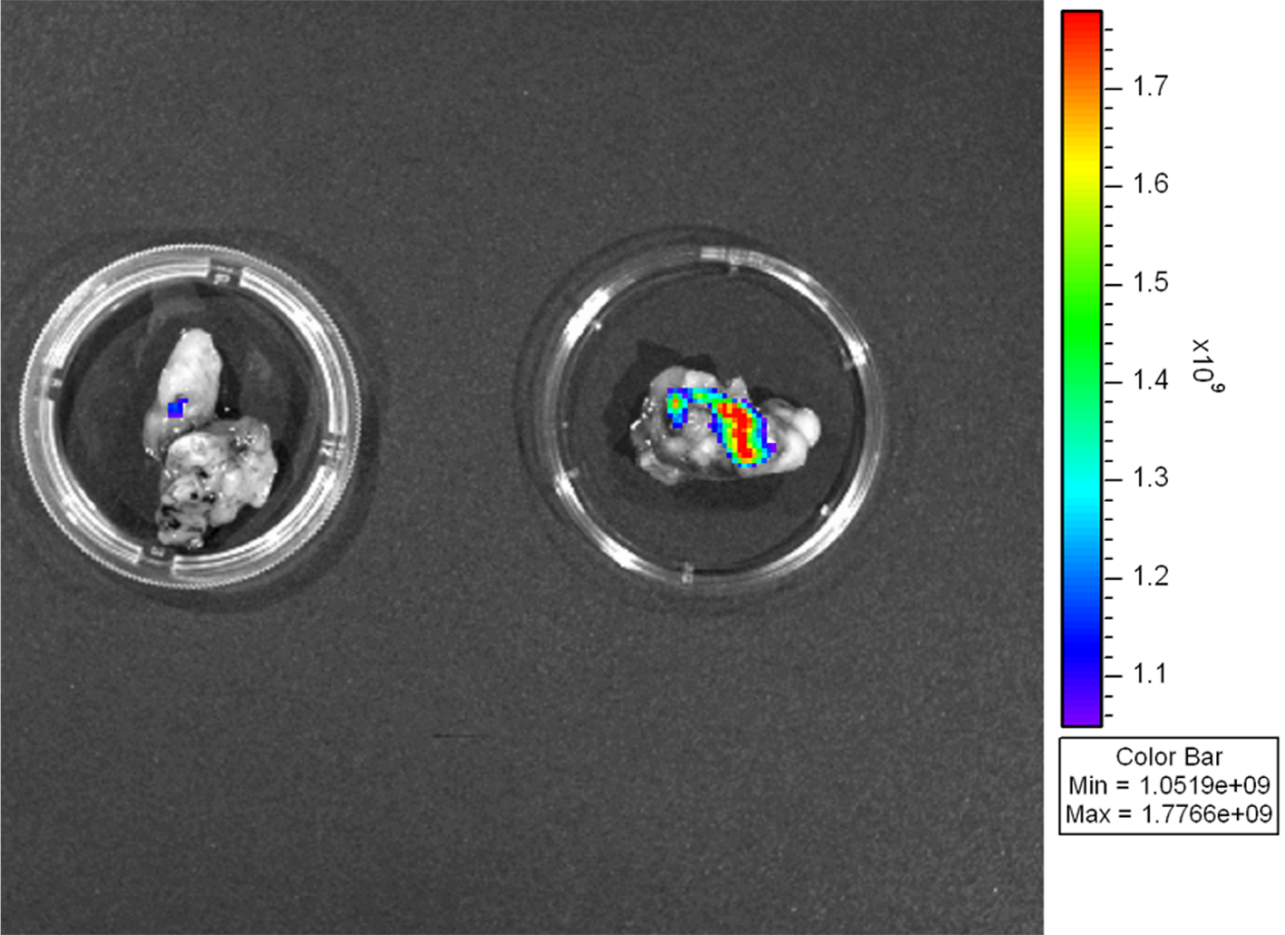
*Ex vivo* NIRF imaging of SKOV3 tumors. NIRF images revealed signals in the dissected tumor masses (right) as compared with in the adjacent control tissue (left). NIRF, near-infrared fluorescence.

**Fig 6:**
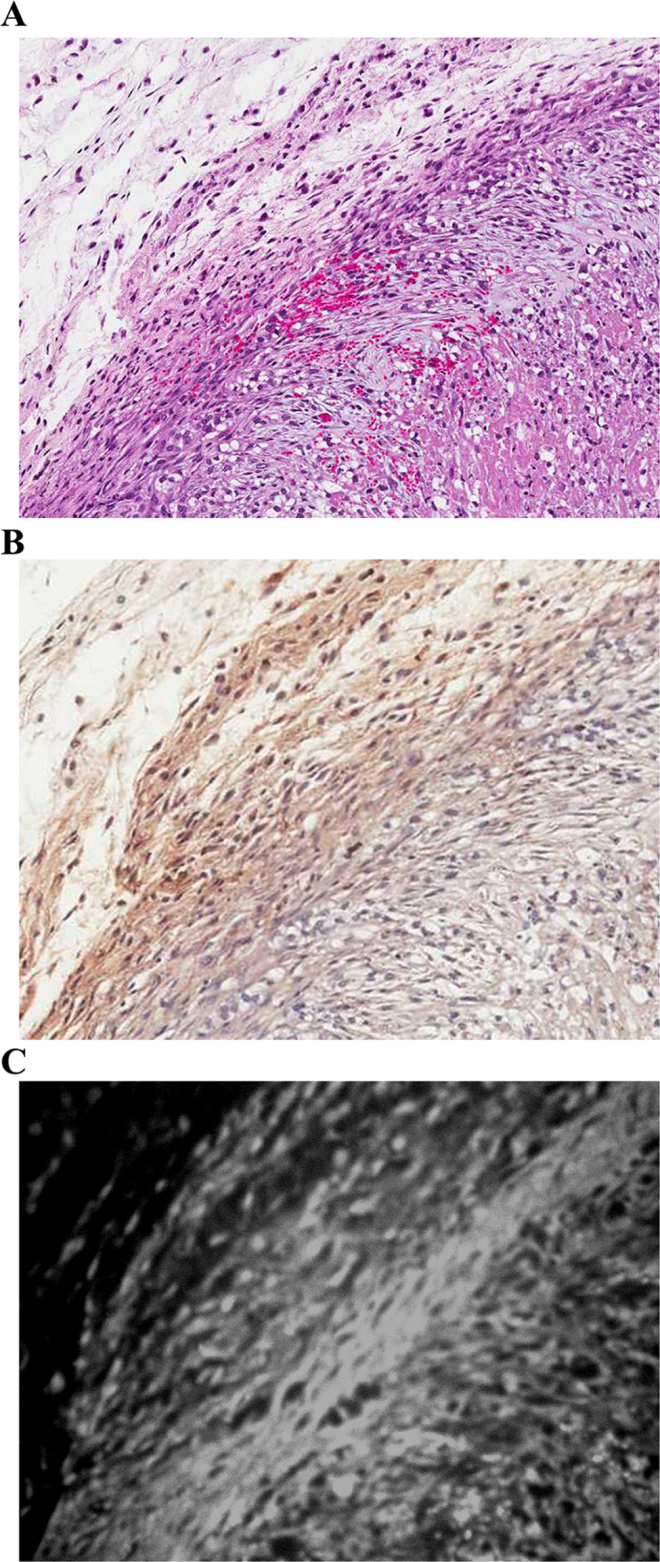
Histological and NIRF analysis of SKOV3 tumor tissues. SKOV3 tumors were analyzed through (A) hematoxylin and eosin staining, (B) immunohistochemistry using an MMP-3 antibody, and (C) NIRF with the MMP-3-sensitive probe. MMP, matrix metalloproteinase; NIRF, near-infrared fluorescence.

As shown in Fig 5, an increased NIRF signal was obtained in the tumor tissue as compared with in the control tissue (normal subcutaneous tissue adjacent to the tumor). Hematoxylin and eosin (H&E) staining of the tumor revealed infiltrating immune cells in the tumor mass and the surrounding connective tissue (Fig 6A). Immunostaining with an MMP-3 antibody revealed a predominantly positive-stained region located between the tumor mass and surrounding connective tissue, which was colocalized with the positive signal obtained by using the MMP-3-sensitive probe and NIRF imaging (Fig 6B and 6C, respectively). Taken together, MMP-3 activity was primarily localized at the boundary of tumor mass and the adjacent connective tissue, an area that was also infiltrated by inflammatory monocytes.

To further understand the in vivo stability and biodistribution of the probe, stability test and biodistribution evaluation were perform. As shown in the stability test (S9 Fig), the probe were mainly distributed to abdomen after 1 hour of injection, and then distributed to kidney 36hr after the injection. For biodistribution evaluation, Individual organs were harvested and further evaluated for fluorescent intensity. The MMP-3-sensitive probe specifically targeted the tumor mass, with signals detected also at liver and kidney (S10 Fig). For the control probe, baseline fluorescent signal was acquired at liver and kidney (S10 Fig), indicating the biodistribution of the target organs during excretion of the probe from the circulation.

## Discussion

Specific imaging tools are required to detect early-stage EOC to improve the survival outcomes for these patients. Because MMPs play critical roles in carcinogenesis as well as cancer invasion and metastasis and are expressed at the early stages of disease progression [7, 14, 15], we synthesized and characterized a novel MMP-3-sensitive probe, and its specificity for MMP-3 activity was examined in both *in vitro* and animal models of EOC. The MMP-3-sensitive probe could detect MMP-3 activity in SKOV3 and WS1 cocultures as well as in SKOV3 tumors *in vivo*, suggesting that it could aid in detecting early-stage EOC.

Lee et al. [28] previously reported the use of NIRF imaging of a gold nanoparticle for the detection of MMP activity; however, this probe was not specific because it could be cleaved by MMP-2, MMP-3, MMP-7, and MMP-13. By contrast, our data demonstrated that the synthesized MMP-3-sensitive probe could specifically detect increased MMP-3 activity not only in cell cultures but also in tumor-bearing mice *in situ*. Immunostaining of the tumors revealed that MMP-3 was clocalized in the probe-binding region, confirming that the MMP-3-sensitive probe could specifically detect MMP-3 activity in the target region. Studies have confirmed that many early-stage cancers express elevated levels of MMP-2, MMP-3, and MMP-9 [7, 14, 15]. Specifically, MMP-3 has been associated with tumor initiation and progression [17, 31, 32], and the loss of MMP-3 was shown to suppress lung metastasis in an *in vivo* model of mammary carcinoma [32]. MMP-2 and MMP-9 are expressed in the malignant epithelia of tumors that have undergone an epithelial-to-mesenchymal transformation [35]. These MMPs are crucial for local invasion of the tumor, angiogenesis, and vascular invasion [36]; thus, they represent potential therapeutic targets [37]. In the present study, early expression of MMP-3 was observed in the SKOV3 and WS1 cocultures, and MMP-3 activity detected by the novel probe peaked at day 5 after the inoculation of SKOV3 cells *in vivo*. Additional studies are required to confirm the early-stage activity of MMP-3 in EOC and its diagnostic and prognostic significance.

Cancer-associated MMP expression and activity originates not only in cancer cells but also in the stromal cells of the surrounding connective tissue. Specifically, fibroblasts and inflammatory cells in the connective tissue may induce MMPs in response to tumor formation, which can provoke the invasion of the malignant epithelial cells [38]. In certain epithelial cancers, host stromal cells expressed most of the up-regulated MMPs during cancer invasion and disease progression [39]. Consistent with these studies, our data demonstrated that SKOV3 cells expressed minimal levels of MMP-3 when cultured alone. However, MMP-3 expression was increased upon cocultivation with WS1 cells. This increase in MMP-3 expression levels also occurred earlier than that observed for MMP-2 and MMP-9 in cocultures. Our pathological analysis demonstrated that both MMP-3 expression and activity were increased at the boundary between the tumor mass and the adjacent connective tissue consisting mainly of fibroblasts; this finding is consistent with another study reporting MMP-3 expression by fibroblasts, endothelial cells, and immunocytes exclusively in the tumor stroma. MMP-3 also exhibited to have a wide range of substrate specificity for various ECM components [11]. The nature of its enzyme activity and expression pattern implies that MMP-3 plays critical role during an early phase of tumor progression [16] when ovarian cancer cells interact with fibroblasts.

Molecular imaging techniques have several advantages over classical imaging techniques as well as in vitro assays. For example, the analysis of both spatial and temporal distributions of a protein or its activity enables to permit a more global analysis [21]. Furthermore, because molecular imaging techniques can be performed in live animals, repeated measurements are possible, thereby decreasing costs [21]. In addition, these measurements are quantitative in nature.

The present study has some limitations that warrant further discussion. First, the specificity of the probe was only tested in a subcutaneous EOC model in animals. Therefore, additional studies are necessary to evaluate its utility in women with EOC. In addition, this particular model may not reflect the complexities of EOC. Furthermore, additional studies analyzing the diagnostic and prognostic value of the early detection of MMP-3 activity in EOC are required.

The development of technologies enabling noninvasive and sensitive detection of early-stage EOC is crucial to improve patient survival. The current study tested a molecular imaging reporter for detecting EOC disease progression *in situ* by identifying MMP-3 activity in both a cell and animal model. The detection of MMP-3 activity at day 5, which subsequently decreased, implies that this molecule may have a “hit-and-run” a role during early-stage tumor growth and suggests that MMP-3 could be a potential molecular target for the early detection of EOC.

## Supporting information

S1 Scheme Synthesis diagram of stromelysine-1-sensitive peptide substrate and control peptide. (TIFF)

S1 Fig Synthesis diagram of the MMP-3 peptide and control peptide. (TIF)

S2 Fig Synthesis diagram of stromelysine-1-sensitive probe and control probe. (TIF)

S3 Fig HPLC chromatogram of stromelysine-1 peptide substrate. The peaks were detected by UV/Vis = 254 nm. A Superose 6 HR 10/30 column was used, eluted with 0.1% formic acid (0.4 ml/ min).
(TIFF)

S4 Fig HPLC chromatogram of stromelysine-1 control peptide substrate. The peaks were detected by UV/Vis = 254 nm. A Superose 6 HR 10/30 column was used, eluted with 0.1% formic acid (0.4 ml/ min).
(TIFF)

S5 Fig UV-Vis spectrum of Cy5.5-s1-PGC probe. (TIFF)

S6 Fig Fluorescence spectrum of Cy5.5-s1-PGC probe 5μM; ex = 670 nm (blue line) and Cy5.5 1μM; ex = 670 nm (red line). (TIFF)

S7 Fig HPLC chromatogram of stromelysine-1 peptide substrate (black) and their digested products by MMP-3 (gray). The peaks were detected by fluorescence detector at ex/em = 485/530 nm. A Supelco RP-C18 column (5μm, 4.6 × 250 mm) was used, eluted with MeOH: 0.1% formic acid = 60: 40 (0.6 ml/min).
(TIF)

S8 Fig HPLC chromatogram of stromelysine-1 control peptide substrate (black) and their digested products by MMP-3 (gray). The peaks were detected by fluorescence detector at ex/em = 485/530 nm. A Supelco RP-C18 column (5μm, 4.6 × 250 mm) was used, eluted with MeOH: 0.1% formic acid = 60: 40 (0.6 ml/min).
(TIF)

S9 Fig Mice were intravenously injected with 200 μL of 2nM MMP-3-sensitive probe via tail vein. Fluorescent images were taken after the injection at various time points. The probe were distributed to abdomen after 1 hour of injection and then distributed to kidney 36hr after the injection.
(TIFF)

S10 Fig Biodistribution of MMP-3 sensitive probe and control probe in ovarian tumor-bearing mice. Biodistribution of MMP-3 sensitive probe (A) and the control probe (B) was investigated at 24 h after injection in ovarian tumor-bearing mice. The ovarian cancer mass showed significant signal intensity in the group of the MMP-3-sensitive probe, whereas no signal in the group of control probe. The spleen, liver and kidney showed mild to marked signal intensity; however, bone, heart, lung, and muscle exhibited no signal. The tissues are oriented as followings: heart, lung, spleen, tumor (left to right) (Top) kidney, muscle, bone, liver (Left to Right) (Bottom).
(TIF)
