# Correction: Optical imaging of ovarian cancer using a matrix metalloproteinase-3-sensitive near-infrared fluorescent probe

**DOI:** 10.1371/journal.pone.0202610

**Published:** 2018-08-14

**Authors:** Kuo-Hwa Wang, Yun-Ming Wang, Li-Hsuan Chiu, Tze-Chien Chen, Yu-Hui Tsai, Chun S. Zuo, Kuan-Chou Chen, Chun Austin Changou, Wen-Fu T. Lai

The second author's name is spelled incorrectly. The correct name is: Yun-Ming Wang. The correct citation is: Wang K-H, Wang Y-M, Chiu L-H, Chen T-C, Tsai Y-H, Zuo CS, et al. (2018) Optical imaging of ovarian cancer using a matrix metalloproteinase-3-sensitive near-infrared fluorescent probe. PLoS ONE 13(2): e0192047. https://doi.org/10.1371/journal.pone.0192047
