# Supplementary figures and images for: Optical imaging of ovarian cancer using a matrix metalloproteinase-3-sensitive near-infrared fluorescent probe
Source: PLoS One. 2018 Feb 1;13(2):e0192047. doi: 10.1371/journal.pone.0192047 (PMC5794152; doi:10.1371/journal.pone.0192047)

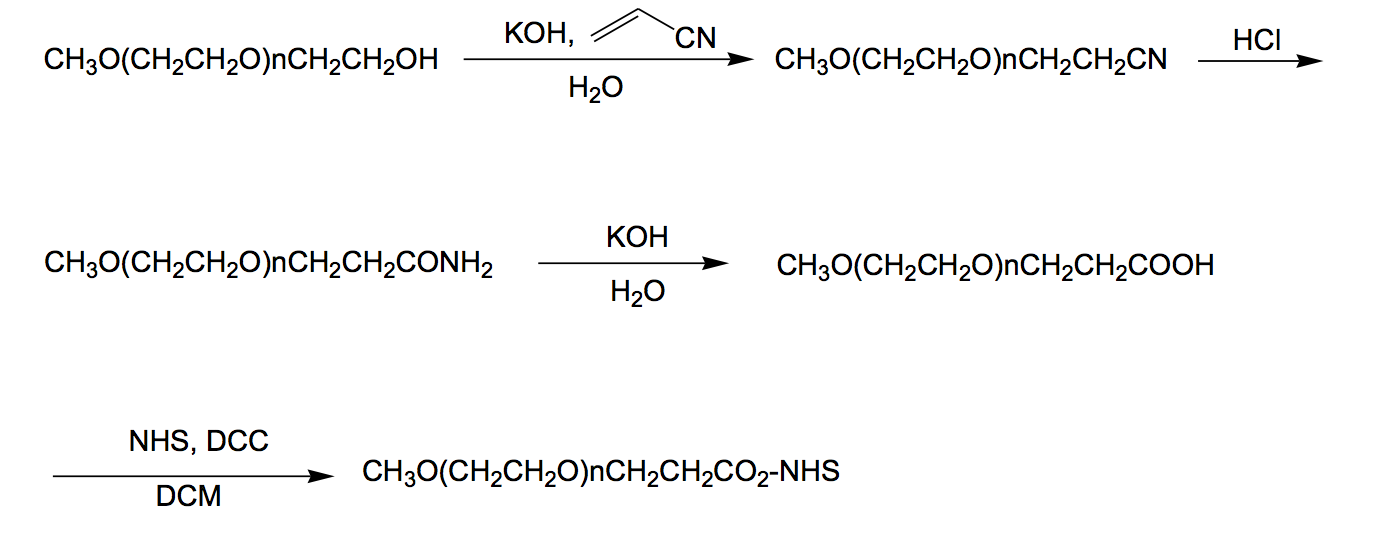

Supplement: S1 Scheme — (TIFF) [file pone.0192047.s001.tiff]

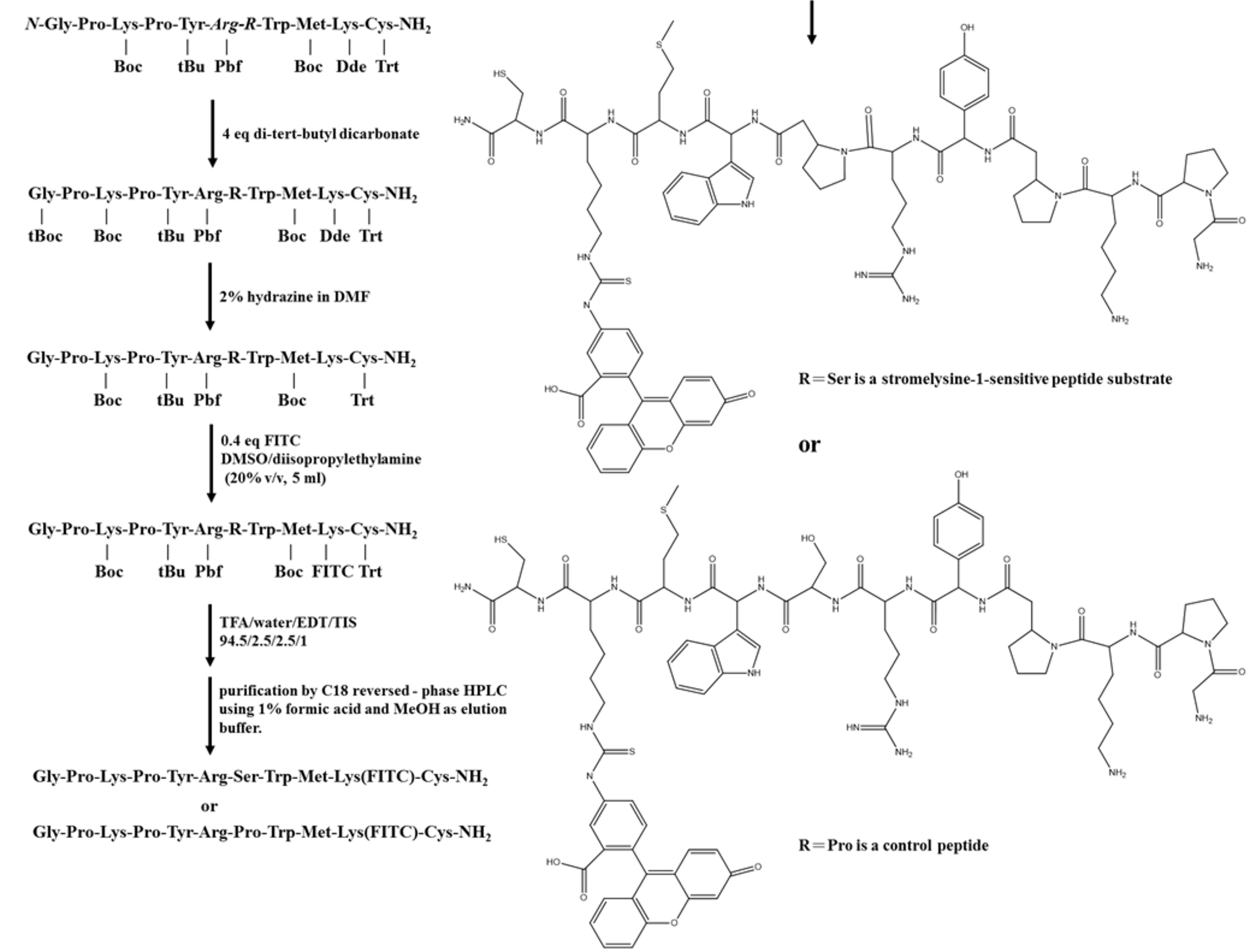

Supplement: S1 Fig — (TIF) [file pone.0192047.s002.tif]

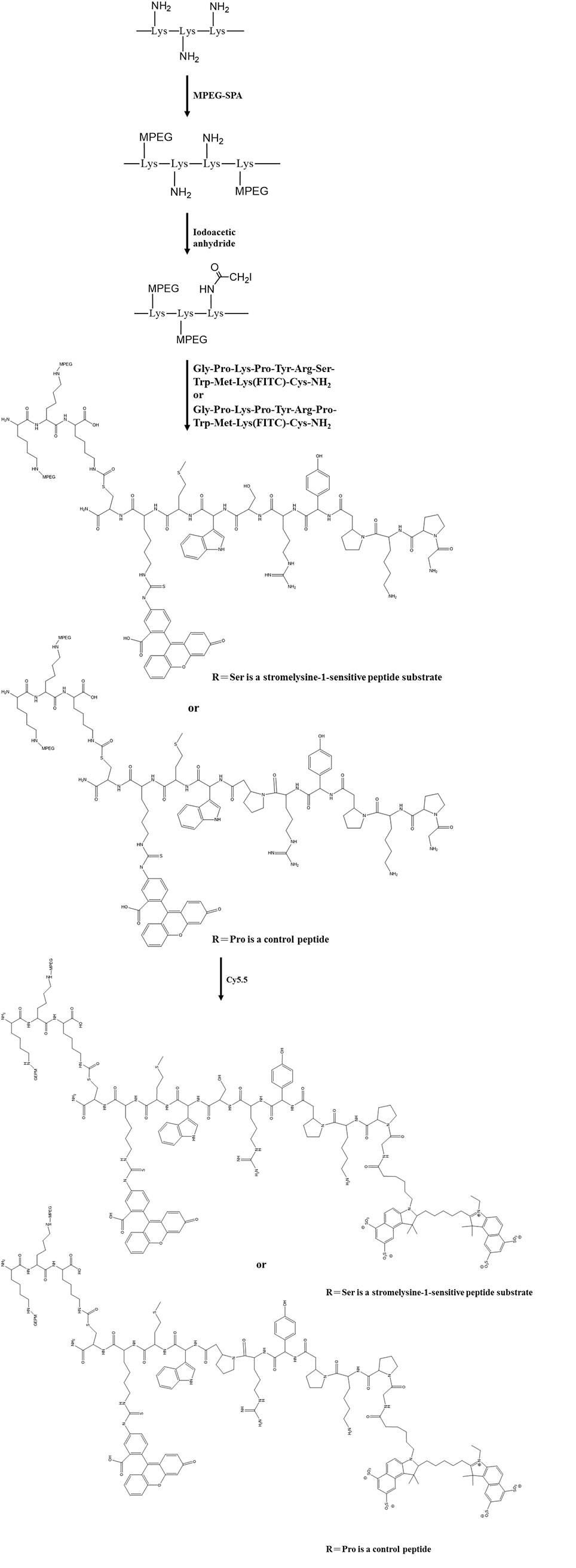

Supplement: S2 Fig — (TIF) [file pone.0192047.s003.tif]

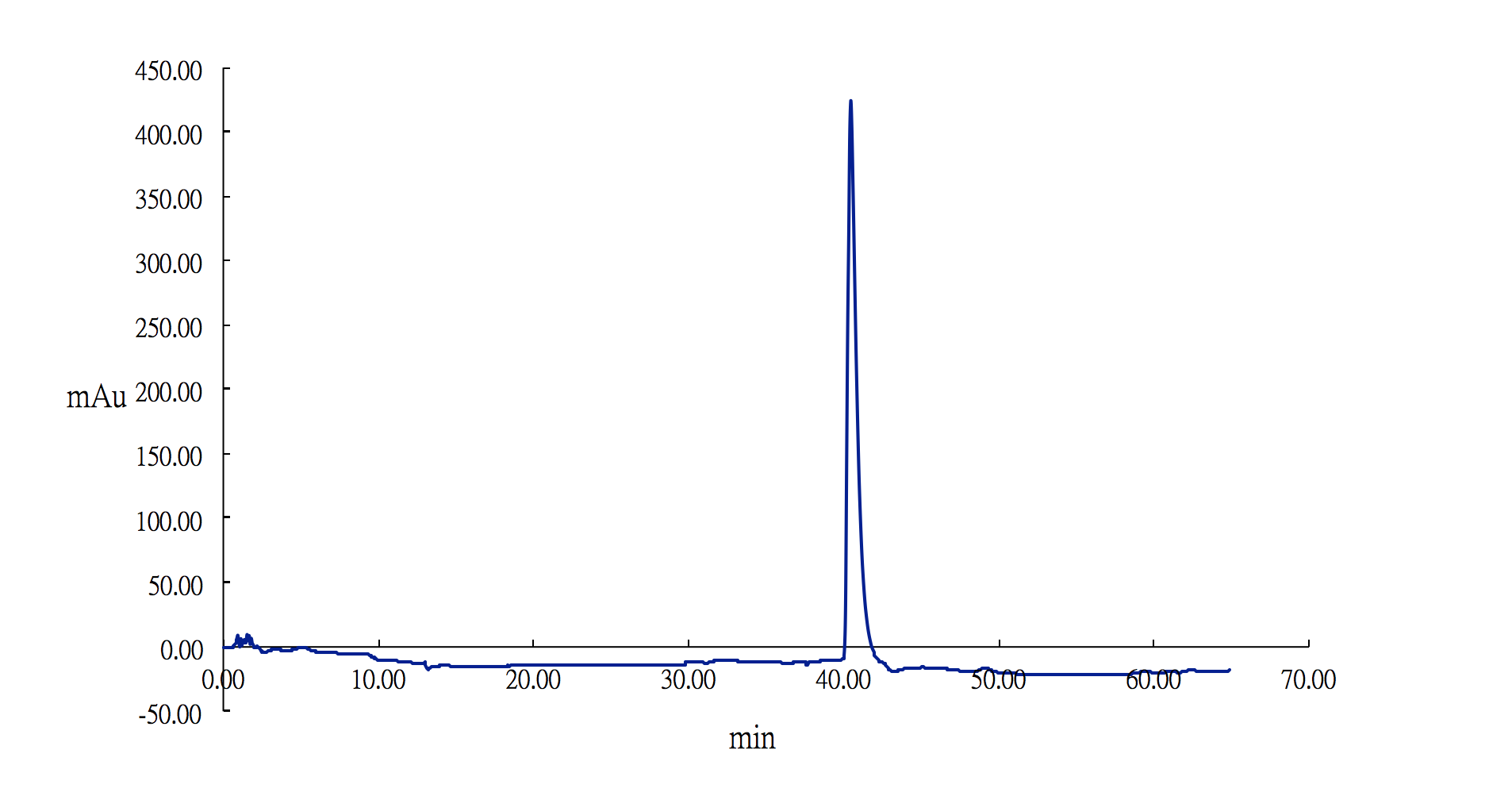

Supplement: S3 Fig — The peaks were detected by UV/Vis = 254 nm. A Superose 6 HR 10/30 column was used, eluted with 0.1% formic acid (0.4 ml/ min). (TIFF) [file pone.0192047.s004.tiff]

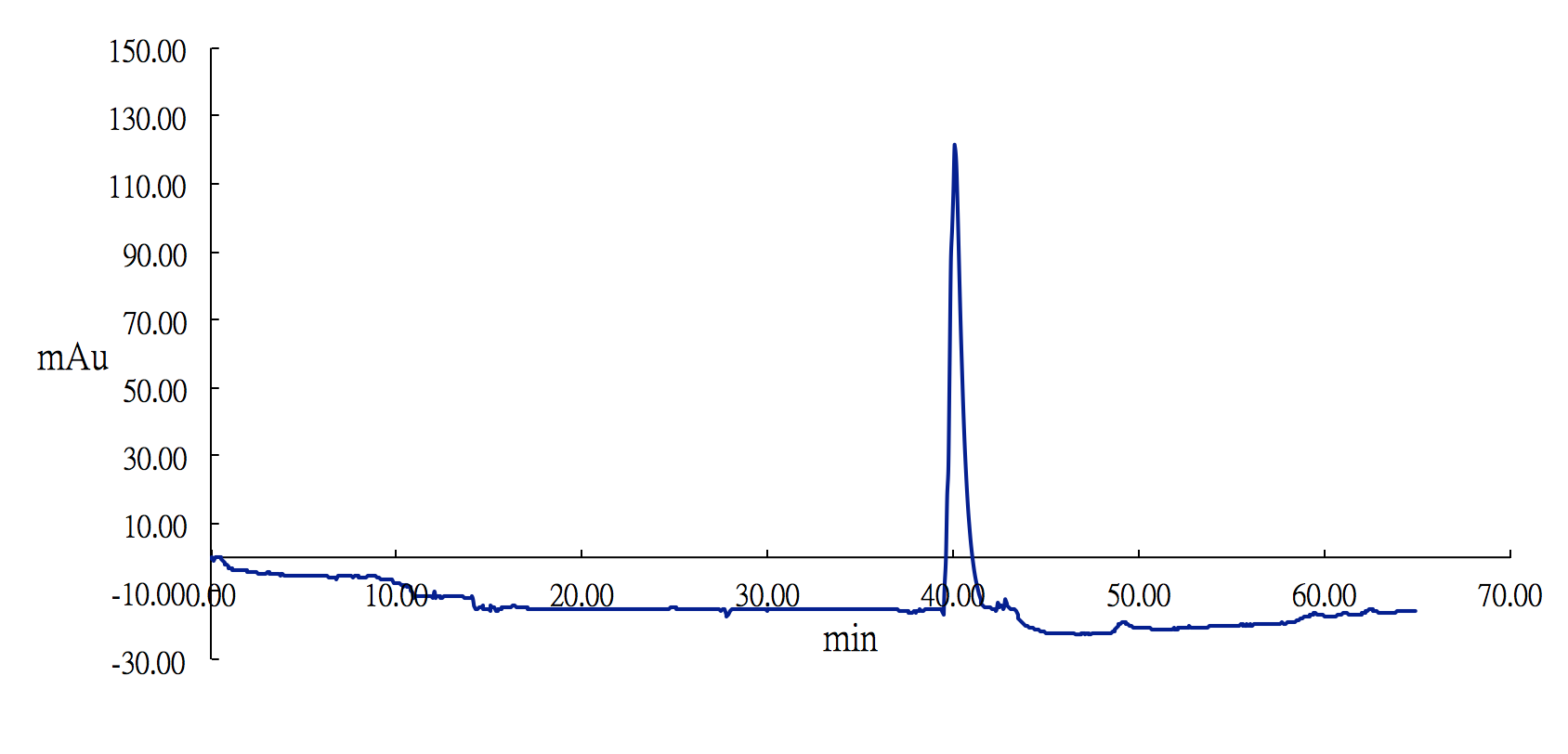

Supplement: S4 Fig — The peaks were detected by UV/Vis = 254 nm. A Superose 6 HR 10/30 column was used, eluted with 0.1% formic acid (0.4 ml/ min). (TIFF) [file pone.0192047.s005.tiff]

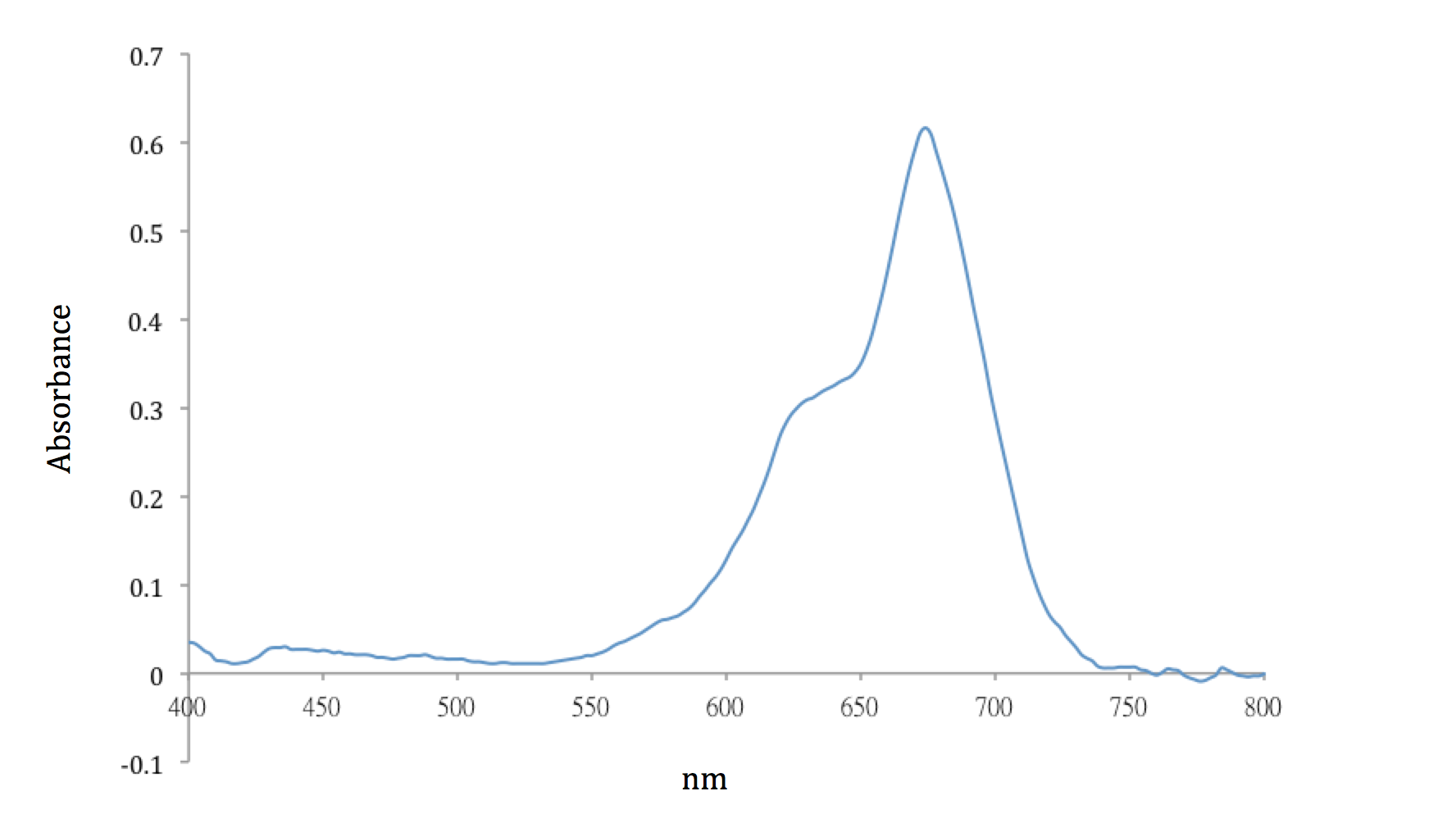

Supplement: S5 Fig — (TIFF) [file pone.0192047.s006.tiff]

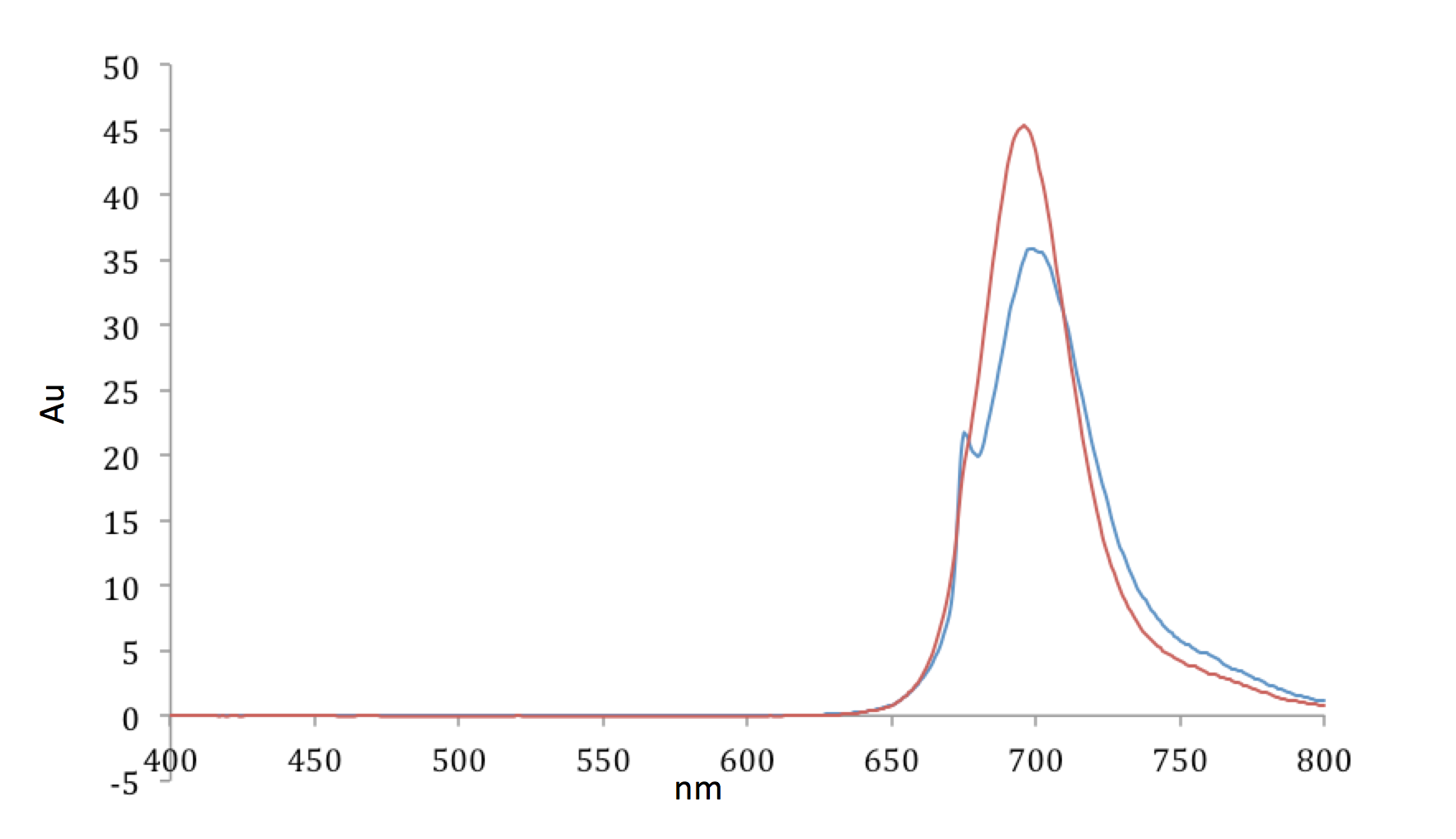

Supplement: S6 Fig — (TIFF) [file pone.0192047.s007.tiff]

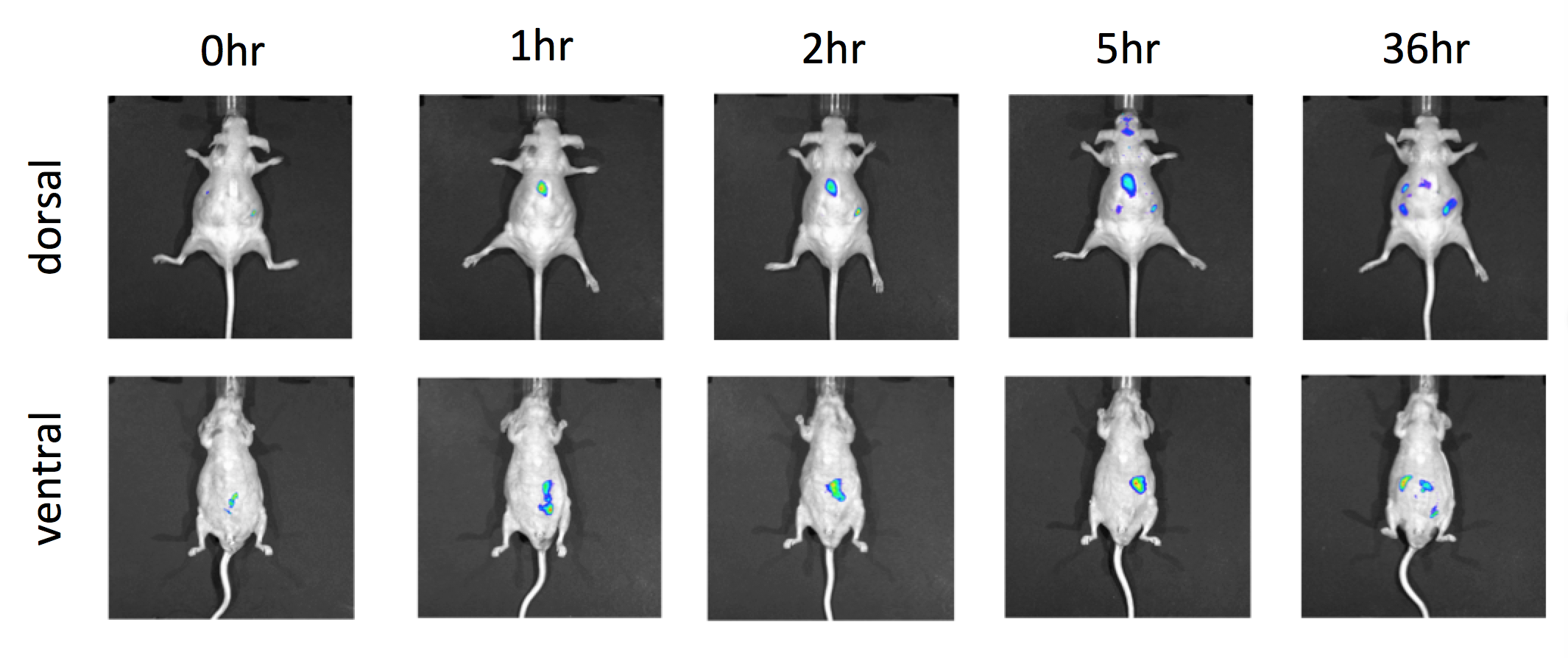

Supplement: S9 Fig — Fluorescent images were taken after the injection at various time points. The probe were distributed to abdomen after 1 hour of injection and then distributed to kidney 36hr after the injection. (TIFF) [file pone.0192047.s010.tiff]

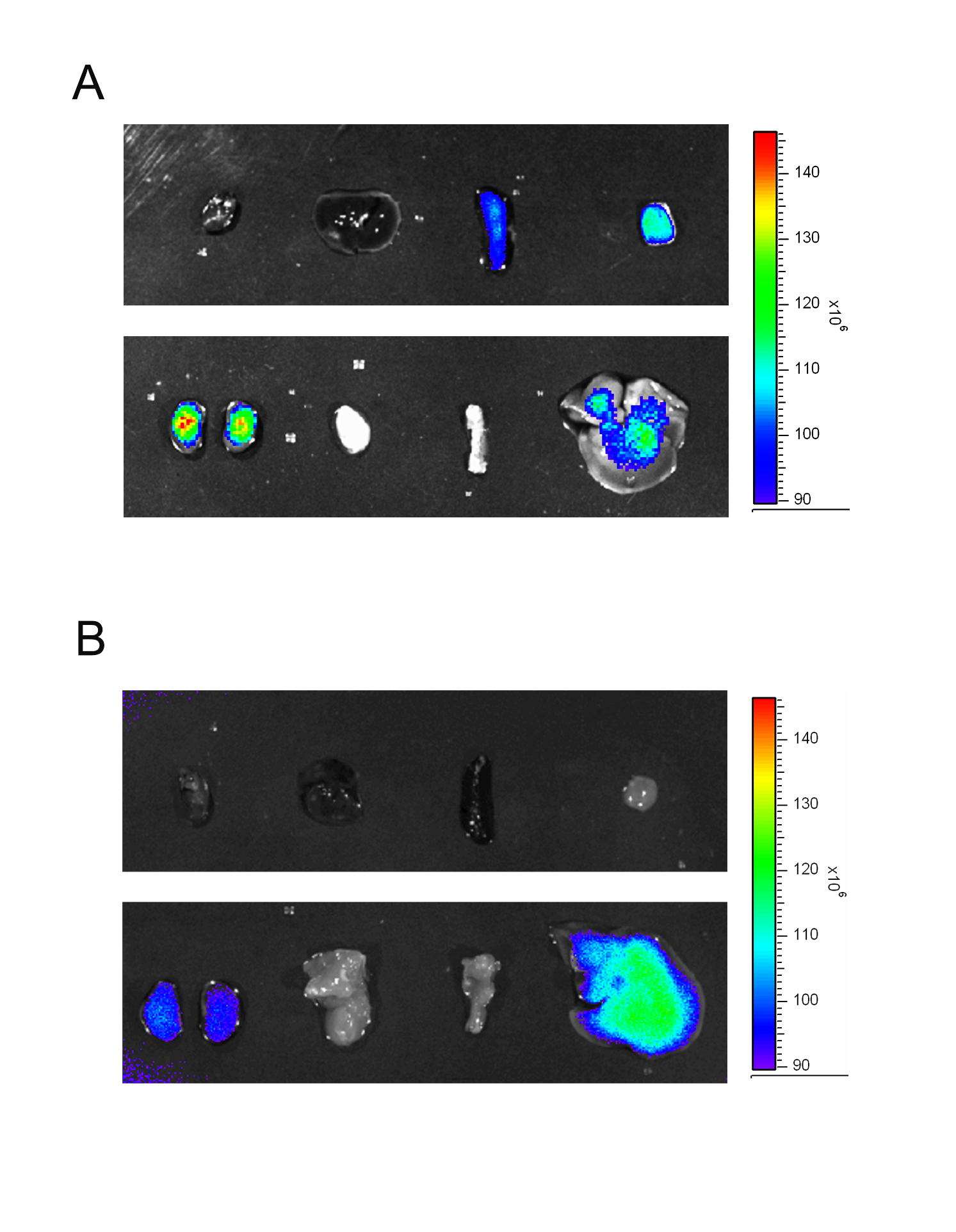

Supplement: S10 Fig — Biodistribution of MMP-3 sensitive probe (A) and the control probe (B) was investigated at 24 h after injection in ovarian tumor-bearing mice. The ovarian cancer mass showed significant signal intensity in the group of the MMP-3-sensitive probe, whereas no signal in the group of control probe. The spleen, liver and kidney showed mild to marked signal intensity; however, bone, heart, lung, and muscle exhibited no signal. The tissues are oriented as followings: heart, lung, spleen, tumor (left to right) (Top) kidney, muscle, bone, liver (Left to Right) (Bottom). (TIF) [file pone.0192047.s011.tif]
